# Diencephalic Syndrome: Clinical Features, Pathophysiology, and Long-Term Outcomes

**DOI:** 10.3390/children13020165

**Published:** 2026-01-24

**Authors:** Antonio Ruggiero, Palma Maurizi, Alberto Romano, Fernando Fuccillo, Dario Talloa, Stefano Mastrangelo, Giorgio Attinà

**Affiliations:** 1 Pediatric Oncology Unit, Fondazione Policlinico Universitario Agostino Gemelli IRCCS, 00168 Rome, Italy; palma.maurizi@unicatt.it (P.M.); alberto.romano@policlinicogemelli.it (A.R.); fernando.fuccillo@unicatt.it (F.F.); dario.talloa@guest.policlinicogemelli.it (D.T.); stefano.mastrangelo@unicatt.it (S.M.); giorgio.attina@policlinicogemelli.it (G.A.); 2 Department of Woman and Child Health and Public Health, Università Cattolica del Sacro Cuore, 00168 Rome, Italy

**Keywords:** diencephalic syndrome, failure to thrive, hypothalamic glioma, optic pathway glioma, cachexia, hypothalamic obesity

## Abstract

**Background/Objectives**: Diencephalic syndrome (DS) is an uncommon pediatric disorder presenting with severe failure to thrive despite adequate caloric intake and preserved linear growth. First characterized by Russell, this condition predominantly affects infants under 12 months and remains diagnostically challenging. **Methods**: We performed a comprehensive literature review examining clinical presentation, underlying pathophysiology, associated pathology, diagnostic approaches, and long-term outcomes of DS. **Results**: DS typically manifests in the first year of life with profound cachexia, normal or increased appetite, preserved height velocity, and characteristic features including hyperactivity, euphorism, and visual pathway involvement. Low-grade gliomas of the hypothalamic–chiasmatic region, particularly pilocytic astrocytomas, represent the predominant underlying pathology. The pathophysiological mechanisms remain incompletely understood but likely involve complex dysregulation of hypothalamic energy homeostasis. While overall survival exceeds 90% at five years, most patients experience significant long-term morbidity including visual impairment, multiple endocrine deficiencies, and hypothalamic obesity. Diagnostic delays averaging 11 months contribute to irreversible complications. **Conclusions**: Early recognition of DS is essential to prevent permanent visual loss and optimize outcomes. Multidisciplinary management incorporating chemotherapy as first-line treatment for underlying gliomas has improved survival while reducing radiation-associated toxicities. However, survivors face substantial lifelong sequelae requiring comprehensive monitoring and intervention. Future research should focus on elucidating precise pathophysiological mechanisms, developing targeted molecular therapies, and improving management of hypothalamic obesity and other late effects.

## 1. Introduction

Diencephalic syndrome represents an unusual cause of infantile failure to thrive, characterized by profound emaciation occurring despite adequate nutritional intake and maintained linear growth [1,2]. Russell provided the first comprehensive description in 1951, establishing diagnostic criteria that remain clinically relevant [3]. The syndrome occurs almost exclusively in association with hypothalamic–chiasmatic gliomas, typically presenting before 12 months of age with a median onset at 6–7 months [2,4].

Beyond severe cachexia, affected children demonstrate distinctive behavioral features including marked hyperactivity inappropriate for their nutritional state and paradoxically euphoric demeanor [3,5]. Visual pathway involvement occurs nearly universally, with nystagmus, strabismus, and progressive optic atrophy contributing to long-term visual morbidity [1,6]. The rarity of DS, with fewer than 200 cases reported over seven decades, combined with nonspecific early symptoms, contributes to delayed diagnosis and accumulated irreversible damage [7].

A comprehensive narrative literature review was conducted to examine clinical manifestations, pathophysiological mechanisms, diagnostic approaches, treatment modalities, and long-term outcomes in children with diencephalic syndrome. Electronic database searches were performed in PubMed/MEDLINE covering publications from January 1951 through December 2024. The starting date corresponds to Russell’s seminal description establishing diagnostic criteria for this condition.

Given the rarity of diencephalic syndrome with fewer than 200 reported cases over seven decades, no exclusion criteria based on study design were applied. Case reports, case series, cohort studies, clinical trials, and expert consensus statements published in English were included. Priority was given to original research articles, large multicenter cohorts, and publications providing detailed clinical, radiological, histopathological, or molecular characterization. Historical landmark publications that shaped current understanding were incorporated regardless of publication date. Publications addressing specific aspects including epidemiology, pathophysiology, associated tumor biology, therapeutic interventions, and survivorship outcomes were systematically reviewed and synthesized to provide comprehensive disease characterization.

### 1.1. Epidemiology and Clinical Presentation

Initial case reports of emaciated infants with hypothalamic tumors appeared during the 1930s, but Russell’s 1951 publication provided the first systematic characterization [3]. Addy and Hudson consolidated understanding in 1972 through a review of 48 cases, while Burr’s 1976 analysis of 72 patients further refined the clinical phenotype [2,8]. True incidence remains uncertain due to rarity and probable underdiagnosis. Males appear to be slightly more frequently affected in most series, though this observation’s significance remains unclear. Cases occur worldwide across diverse populations [1,7]. Age distribution shows a marked concentration in early infancy, with approximately 90% presenting before 24 months [2,4,8]. This characteristic pattern may reflect developmental vulnerability of the immature hypothalamus to pathological compression [1].

Severe wasting with marked subcutaneous fat depletion occurring alongside normal or minimally reduced caloric intake and preserved linear growth velocity defines DS [1,2]. This paradoxical growth pattern distinguishes the condition from typical failure to thrive. Cachexia can reach extreme degrees, with weight-for-age falling below the first percentile despite documented adequate intake [4,8]. Parents commonly report a voracious appetite and normal feeding behavior despite progressive weight loss [3]. However, appetite patterns are variable, with parents reporting a poor appetite in some cases, normal appetite in others, and excessive appetite in a minority [2]. Vomiting occurs frequently at presentation and may contribute to diagnostic confusion with gastrointestinal pathology.

The behavioral profile warrants attention. Affected children exhibit striking hyperactivity paradoxical to their emaciated state, appearing energetic and socially engaged [3,5]. The euphoric affect contrasts markedly with the withdrawn, apathetic demeanor typical of malnutrition from other causes. Additional features include cutaneous pallor without anemia, hypoglycemia, hypotension, temperature instability, excessive sweating, and altered sleep–wake cycles [4,5,9].

Neuro-ophthalmological abnormalities occur in nearly all cases [1,6]. Nystagmus, present in most patients, frequently represents the initial symptom prompting evaluation [6]. The pattern varies but commonly demonstrates pendular or rotatory characteristics. Strabismus occurs frequently [6,10]. Visual acuity assessment poses challenges in young infants, but objective testing often reveals significant impairment [11]. Optic disc pallor or frank atrophy becomes evident on funduscopic examination as the disease progresses [6,11]. Visual field defects follow predictable patterns based on tumor location. Bitemporal hemianopsia characterizes chiasmal involvement, while more complex patterns associate with extensive hypothalamic extension [11,12]. Progressive visual loss in untreated cases underscores the importance of prompt diagnosis. Hydrocephalus develops in approximately 40–50% of cases, resulting from cerebrospinal fluid pathway obstruction. Clinical signs including irritability, lethargy, and macrocephaly may predominate in some presentations. Motor abnormalities beyond hyperkinesia include hypotonia in younger infants and later-developing spasticity or hemiparesis [1]. Developmental delay or regression may occur across motor, language, and cognitive domains. Seizures occur in a subset of patients [13,14,15]. Hypothalamic involvement produces diverse endocrine abnormalities at diagnosis or during disease evolution [16,17]. Growth hormone (GH) dynamics demonstrate complexity. While linear growth velocity remains preserved initially, growth hormone levels may be paradoxically elevated with abnormal glucose-mediated suppression, suggesting partial GH resistance [2,18,19]. Thyroid axis dysfunction manifests variably, with central hypothyroidism occurring more commonly with progressive hypothalamic damage [17,20]. Adrenal insufficiency requires recognition and management [17,21]. Diabetes insipidus develops in a significant minority, particularly with extensive hypothalamic involvement or following neurosurgical intervention [1,17]. Disorders of pubertal development occur frequently in long-term survivors, including central precocious puberty [1,16,22] or hypogonadotropic hypogonadism [22,23].

### 1.2. Diagnostic Challenges

DS remains a frequently overlooked etiology of infantile failure to thrive. The primary diagnostic difficulty stems from the fact that weight loss in infants typically results from gastrointestinal pathology rather than hypothalamic tumors [24]. Key clinical indicators suggesting DS include an absence of gastrointestinal symptoms and preservation of normal linear growth rate [2,24]. The average diagnostic delay of approximately 11 months from symptom onset represents a critical window during which irreversible visual and neurological damage accumulates [2,8]. Factors contributing to delayed diagnosis include the rarity of the condition, lack of clinician familiarity, attribution of symptoms to more common diagnoses, and nonspecific early manifestations [8,25]. The diagnosis of DS relies primarily on clinical criteria rather than specific quantitative thresholds. While formal diagnostic criteria have not been universally established, DS is generally characterized by the following: (1) weight-for-age below the third percentile or weight loss crossing two or more major percentile lines despite adequate caloric intake; (2) preserved linear growth velocity (height-for-age typically within normal range); (3) characteristic behavioral features including hyperactivity and euphoric demeanor; and (4) neuroimaging evidence of hypothalamic–chiasmatic pathology. Quantitative assessments, when available, may include the following: body mass index (BMI) significantly below age-appropriate norms (typically <−2 standard deviations), documented caloric intake adequate for age and weight, and metabolic studies demonstrating elevated resting energy expenditure. However, the variability in clinical presentation and lack of pathognomonic laboratory findings contribute to diagnostic challenges and delays.

The differential diagnosis encompasses numerous conditions presenting with failure to thrive in infancy. Gastroesophageal reflux disease, food allergies, celiac disease, cystic fibrosis, and metabolic disorders warrant consideration [26]. Distinguishing features favoring DS include preserved linear growth velocity, absence of gastrointestinal symptoms, characteristic behavioral profile, and presence of neuro-ophthalmological signs (Table 1) [2,26]. Other central nervous system (CNS) pathologies including hydrocephalus from non-tumoral causes, craniopharyngioma, and hypothalamic hamartoma may occasionally mimic DS [27]. Additionally, rare cases of pseudo-diencephalic syndrome associated with brainstem tumors have been reported, presenting with failure to thrive but typically lacking the characteristic hyperactivity and hypermetabolic features of classic DS [26]. Genetic syndromes affecting hypothalamic development, such as septo-optic dysplasia, require differentiation [28].

### 1.3. Pathophysiology

The pathogenesis underlying cachexia in DS remains incompletely elucidated. Early investigations identified paradoxically elevated growth hormone levels with an abnormal response to glucose loading, suggesting partial GH resistance [4,18]. Excessive secretion of β-lipotropin, a peptide with lipolytic properties, has been postulated to increase adipose tissue breakdown [4]. The growth hormone resistance hypothesis proposes that tumor-induced alterations in hypothalamic regulation lead to excessive GH secretion with impaired peripheral responsiveness [18,19]. Supporting this theory, IGF-1 levels in some DS patients demonstrate dissociation from GH levels [19,29], though this finding has not been consistent across all cohorts [29]. Whether DS reflects an anorexic state or a hypermetabolic condition with increased energy expenditure remains unresolved [18]. Some evidence suggests an enhanced metabolic rate contributes to the cachectic phenotype. Indirect calorimetry studies in limited patient cohorts have demonstrated an elevated resting energy expenditure relative to body mass [5,30]. Metabolic studies have revealed abnormalities in substrate utilization, with preferential fat oxidation and relative protein sparing [30], differing from typical starvation responses [30,31].

Hypothalamic nuclei play crucial roles in appetite regulation, satiety signaling, and energy balance [32]. Tumor involvement of regulatory centers could disrupt normal appetite control mechanisms. Paradoxically, many DS patients demonstrate a normal or increased appetite [3,5]. Leptin levels show variable patterns, with some demonstrating inappropriately low levels for their degree of adiposity [33]. Hypothalamic control of autonomic nervous system function influences metabolic rate, thermogenesis, and substrate mobilization. Dysregulation of sympathetic tone could potentially increase energy expenditure [34]. The hyperactivity characteristic of DS patients may reflect an altered autonomic balance [34]. Clinical observations demonstrating weight gain following tumor treatment and rapid weight loss upon recurrence support a direct causal relationship between the neoplastic process and cachexia development [24].

### 1.4. Associated Pathology

DS occurs almost exclusively in association with space-occupying lesions of the hypothalamic–optic chiasm region, predominantly represented by low-grade gliomas [1,35]. Pilocytic astrocytoma constitutes the most common histological subtype, accounting for approximately 70–80% of DS-associated tumors [1,36]. Pilomyxoid astrocytomas, considered a more aggressive variant, represent another significant proportion [36,37]. Gangliogliomas occur less frequently [38]. Rarely, higher-grade astrocytomas may present with DS, though these aggressive histologies portend a worse prognosis [39]. Tumors associated with DS typically exhibit substantial volume and involve the anterior hypothalamus [1,35]. The anterior hypothalamic involvement appears critical in developing the syndrome’s characteristic features. Volumetric analyses demonstrate that DS tends to occur with larger tumors, suggesting a threshold effect [40]. Histopathological examination of DS-associated Pilocytic Astrocytomas (PAs) reveals typical features including biphasic architecture with compact piloid areas alternating with loose microcystic regions, Rosenthal fibers, eosinophilic granular bodies, and low mitotic activity [41].

Advances in molecular characterization of pediatric low-grade gliomas have revealed distinct genetic alterations. MAPK pathway activation represents a unifying feature across most pediatric low-grade gliomas (LGGs) [42]. BRAF alterations constitute the most common molecular abnormality. BRAF-KIAA1549 fusion occurs in approximately 60–70% of posterior fossa pilocytic astrocytomas but less frequently in supratentorial locations [42,43]. The BRAF V600E point mutation occurs in approximately 10–20% of pediatric LGGs, with a higher frequency in certain locations including the cerebellum and diencephalon [42,44]. FGFR1 alterations occur in a subset of pediatric LGGs, particularly pilomyxoid astrocytomas and diffuse astrocytomas arising in the midline [45]. Understanding the molecular underpinnings has important therapeutic implications. MEK inhibitors, which target downstream effectors of BRAF activation, and selective BRAF V600E inhibitors have demonstrated efficacy in clinical trials, offering potential alternatives to conventional chemotherapy [46,47].

NF1 represents an important consideration, as approximately 50–60% of optic pathway gliomas occur in patients with this genetic syndrome [48]. NF1 results from heterozygous loss-of-function variants in the NF1 gene encoding neurofibromin. The NF1 clinical spectrum demonstrates remarkable variability [49,50]. Optic pathway gliomas develop in approximately 15–20% of children with NF1 [51]. Interestingly, DS manifests exceptionally rarely in NF1-associated optic pathway gliomas. When it does occur, the median age of presentation (approximately 4.7 years) exceeds that of sporadic cases [16]. This age difference holds clinical implications, as an older age at presentation may facilitate diagnosis. The biological basis for reduced DS incidence in NF1-associated optic pathway gliomas remains speculative. NF1-associated tumors typically demonstrate more indolent behavior with slower growth rates and a lower progression risk [52]. DS presentation during follow-up of a child with an established NF1 and optic pathway glioma represents an unusual occurrence, as the syndrome nearly universally manifests as an initial presenting feature. This suggests that acute or subacute development of significant hypothalamic dysfunction, rather than chronic indolent tumor growth, may be required for DS manifestation [53]. Anatomically, NF1-associated optic pathway gliomas more commonly involve bilateral optic nerves and multiple locations, whereas sporadic tumors demonstrate predilection for central chiasmatic and hypothalamic locations [54,55].

## 2. Management

### 2.1. Diagnostic Approach

CNS tumors must be considered in all children presenting with severe, unexplained growth failure accompanied by preserved linear growth rate [2]. The clinical association of cachexia, normal height velocity, and absence of gastrointestinal symptoms should prompt neuroimaging evaluation. Initial diagnostic workup should include a comprehensive history, and a detailed physical examination with anthropometric measurements including head circumference, neurological assessment, and ophthalmological evaluation. Laboratory studies should exclude metabolic and endocrine causes. Brain MRI with and without contrast, including dedicated imaging of the hypothalamic–pituitary region and visual pathways, is essential. Ophthalmological examination including visual acuity testing, visual field assessment, and fundoscopy should be performed. Endocrine evaluation including thyroid function, cortisol, and growth hormone axis assessment is indicated [56]. MRI represents the imaging modality of choice for diagnosing and characterizing DS-associated tumors. Typical findings include a hypothalamic–chiasmatic mass demonstrating T1 hypo-intensity or iso-intensity, T2 hyper-intensity, and variable contrast enhancement [57]. Pilocytic astrocytomas classically exhibit heterogeneous signal characteristics reflecting their biphasic architecture, with cystic components demonstrating fluid signal intensity and solid portions showing variable enhancement patterns [57,58]. Pilomyxoid astrocytomas may demonstrate more homogeneous signal characteristics with less cystic change [37,59]. Advanced MRI techniques including diffusion-weighted imaging, magnetic resonance spectroscopy, and perfusion imaging provide additional characterization [60]. The relationship between tumor volume and DS development has been investigated, with larger volumes generally associated with syndrome manifestation [40]. Serial volumetric analysis provides valuable information regarding tumor growth dynamics and treatment response [61].

If MRI reveals a hypothalamic–chiasmatic mass lesion consistent with a low-grade glioma, complete neuraxis imaging should be obtained to exclude disseminated disease [35,62]. Leptomeningeal dissemination, while uncommon, occurs in a significant minority of DS cases, with the HIT-LGG-1996 study reporting dissemination in approximately 16% of infants with hypothalamic–chiasmatic glioma and DS [63]. The incidence may reach 26% when considering all ages. Disseminated disease at presentation represents a negative prognostic factor and influences treatment decisions. Complete neuraxis imaging at diagnosis is therefore strongly recommended, particularly in young infants presenting with DS. The role of biopsy remains individualized. In children with NF1, clinical and radiological diagnosis without tissue confirmation may be appropriate [62]. In sporadic cases, particularly when imaging features suggest potentially atypical histology, biopsy may be warranted [62,63,64].

### 2.2. Pathological Classification

The original Dodge classification, proposed in 1958, categorized optic pathway gliomas into three stages based on anatomical involvement: stage 1 (optic nerve only), stage 2 (chiasm with or without optic nerve involvement), and stage 3 (hypothalamus or adjacent structures) [65]. The modified Dodge classification provides more detailed anatomical description based on modern MRI capabilities [54]. This enhanced classification enables more precise prognostic stratification, with central chiasmatic and hypothalamic involvement portending worse visual outcomes and higher treatment requirements [54,66]. All DS-associated tumors involve the hypothalamus.

### 2.3. Treatment Principles

Optimal care requires coordination among pediatric oncologists, neurosurgeons, endocrinologists, ophthalmologists, nutritionists, and developmental specialists [67,68]. While the deep hypothalamic location of DS-associated tumors generally precludes complete surgical resection, in selected cases with severe DS or rapidly progressive visual deterioration, early surgical debulking may be considered to achieve rapid clinical improvement and preserve visual function, particularly when tumor location permits safe resection. However, the risks of surgical morbidity, including potential hypothalamic injury and endocrinopathies, must be carefully weighed against potential benefits, and surgery is generally reserved for cases where medical treatment alone is insufficient or when tissue diagnosis is required. Surgical interventions primarily address symptomatic hydrocephalus through cerebrospinal fluid diversion procedures or obtain tissue for diagnosis through stereotactic biopsy [13,64].

Contemporary management strategies emphasize medical treatment as the primary intervention. Chemotherapy has emerged as a first-line therapy, particularly in young children where radiation avoidance represents a priority [68].

Carboplatin–vincristine combinations represent current standard approaches in many centers [68]. Alternative regimens including vinblastine monotherapy have shown promise [69,70]. Given the nearly universal visual impairment in DS patients, the use of ototoxic agents such as cisplatin should be avoided when possible to prevent compounding sensory deficits with hearing loss. Contemporary chemotherapy regimens achieve objective response rates of approximately 25% with overall response rates (including stable disease) of 79% for the primary tumor and similar responses for disseminated lesions when present. Clinical stabilization or improvement occurs in the majority of patients during treatment. However, progression-free survival remains limited, with 5-year PFS ranging from 6–10% in patients with dissemination and 10–40% overall depending on age and disease characteristics [71,72]. Most children with DS require multiple lines of treatment over their disease course. Emerging targeted therapies directed against specific molecular alterations offer potential treatment advances. MEK inhibitors including selumetinib and trametinib have demonstrated efficacy in clinical trials [46,73]. BRAF V600E-specific inhibitors combined with MEK inhibition show activity in V600E-mutated tumors [46]. Historically, radiation therapy represented the standard treatment, with doses of 45–54 Gy achieving tumor control in the majority of patients [74]. However, recognition of significant late effects, particularly in young children, has led to paradigm shifts favoring chemotherapy as initial treatment with radiation reserved for salvage scenarios [74]. Late effects include vasculopathy with stroke risk, progressive cognitive decline, endocrinopathies, and secondary malignancy risk [74,75]. When radiation becomes necessary, modern techniques including proton therapy may reduce the dose to normal structures [76,77].

Weight restoration occurs following effective tumor treatment, validating the causal relationship between the neoplasm and cachexia [24]. Nutritional support plays a crucial adjunctive role. Many patients require supplemental feeding via nasogastric tube or gastrostomy to achieve adequate caloric intake during the period before the tumor responds to treatment [1,78].

A resume of clinical presentation and management of DS is provided in Table 1.

## 3. Long-Term Outcomes

### 3.1. Visual Prognosis

Despite advances in treatment, visual outcome in children with DS remains generally unfavorable [1]. Visual outcomes vary considerably, ranging from mild visual impairment to complete blindness, with most patients experiencing significant residual deficits even after successful tumor control [6,11]. Factors influencing visual prognosis include age at diagnosis, duration of symptoms before treatment initiation, extent of optic pathway involvement, and degree of optic atrophy at presentation [11,79]. Children diagnosed at younger ages, particularly those under 12 months, face an increased risk of poor visual outcomes [79]. Prolonged diagnostic delays allow progressive optic nerve damage. The natural history demonstrates progressive deterioration without treatment, with stabilization or modest improvement possible following successful therapy. However, true visual recovery remains uncommon.

### 3.2. Endocrine Sequelae

Hypothalamic involvement inherently predicts significant endocrine morbidity. Common long-term endocrinopathies include thyroid-stimulating hormone deficiency, central precocious puberty, growth hormone deficiency, adrenocorticotropic hormone deficiency, central diabetes insipidus, gonadotropin deficiency, and hypothalamic obesity [1,16].

Central hypothyroidism represents the most prevalent endocrine complication, occurring in 60–70% of patients in some series [1,17]. Thyroid hormone replacement therapy proves essential [80]. Growth hormone deficiency develops in 20–40% of survivors, though the frequency varies with the treatment modality [1,17,81]. Growth hormone therapy does not appear to increase recurrence risk in patients with stable or regressed low-grade gliomas [82,83]. Central precocious puberty affects 40–50% of patients in longitudinal follow-up studies [1,22]. The mechanism remains incompletely understood but may involve the loss of inhibitory control over gonadotropin-releasing hormone secretion [84]. Treatment with gonadotropin-releasing hormone analogs suppresses pubertal progression [84,85]. Conversely, hypogonadotropic hypogonadism with pubertal delay or arrest develops in some patients [22,23], necessitating sex steroid replacement therapy [86]. Central adrenal insufficiency occurs in 20–30% of patients and requires lifelong glucocorticoid replacement [17,21]. Recognition proves critical, as an acute adrenal crisis during physiological stress can be life-threatening [87]. Central diabetes insipidus develops in approximately 20–35% of patients [1,88]. Desmopressin replacement effectively manages symptoms [89].

Hypothalamic obesity, perhaps the most challenging long-term complication, develops in 30–50% of long-term survivors [1,90]. This condition differs fundamentally from typical obesity, arising from disrupted energy balance regulation [90]. The pathophysiology involves impaired satiety signaling, altered autonomic function favoring energy storage, and reduced energy expenditure. Patients experience rapid weight gain, particularly following the resolution of DS-associated cachexia. Conventional weight management strategies demonstrate limited efficacy [91,92]. Management approaches incorporate nutritional counseling, structured physical activity programs, behavioral interventions, and in some cases, pharmacotherapy [90,91,92,93,94,95,96,97]. The metabolic complications including insulin resistance, type 2 diabetes mellitus, dyslipidemia, hypertension, and obstructive sleep apnea significantly impact long-term health. Children with severe hypothalamic obesity should undergo polysomnography to screen for obstructive sleep apnea and, if detected, it can be managed with continuous positive airway pressure therapy or other interventions, potentially improving quality of life and reducing cardiovascular morbidity.

### 3.3. Neurological and Cognitive Impact

Beyond endocrine dysfunction, survivors frequently experience neurological sequelae including seizures, motor deficits, language disturbances, learning difficulties, and psychomotor delay [1]. Seizures occur in 15–25% of patients during the disease course [15]. Motor impairments range from mild hemiparesis to severe spastic quadriplegia [97]. Cerebellar dysfunction manifesting as ataxia, dysmetria, and intention tremor occurs with posterior extension [93].

Cognitive function in DS survivors demonstrates considerable heterogeneity, with outcomes ranging from normal intelligence to significant intellectual disability [14,97]. Multiple factors influence cognitive outcomes including age at diagnosis, tumor location and extent, hydrocephalus severity, treatment modality, and socioeconomic factors [98,99]. A young age at diagnosis confers increased vulnerability [99]. Specific domains frequently affected include processing speed, working memory, executive functions, and visual–spatial skills [96]. Cognitive deficits may progress over time even after tumor control [100,101]. Academic achievement difficulties manifest in substantial proportions of survivors [102]. Behavioral difficulties including attention problems, anxiety, depression, and social skill deficits occur more frequently in survivors [103]. The hyperactivity and euphoric affect characteristics of acute DS typically resolve with tumor treatment but may be replaced by different behavioral challenges including apathy, emotional lability, and irritability [103,104]. Quality of life assessments reveal significant impairments across multiple domains.

### 3.4. Survival Outcomes

Overall survival in DS-associated optic pathway gliomas demonstrates significant age-dependent variability. While contemporary series of older children report 5-year overall survival rates of 90–100%, outcomes in infants are substantially worse. In the HIT-LGG-1996 study, children diagnosed under 1 year of age with DS had a 10-year overall survival rate of only 42.7%, highlighting the particularly aggressive nature of tumors presenting with DS in early infancy [71]. Historical series, such as Addy’s 1972 publication, reported mortality in 24 of 48 patients (50%), though improved contemporary treatment strategies have enhanced survival, particularly in older children [2]. The marked difference in survival between infants and older children reflects both tumor biology and the vulnerability of the developing brain to therapeutic interventions.

However, progression-free survival demonstrates considerably less favorable outcomes, with most patients experiencing at least one tumor progression event [1,18]. Five-year progression-free survival rates range from 10 to 40% across published series [1,18].

Extended follow-up beyond 5 years reveals that survival curves continue to decline over time without reaching a plateau. Deaths occurring more than 10 years after diagnosis, attributed to progressive tumor growth or treatment complications, underscore the chronic nature of this disease. This pattern emphasizes the inadequacy of 5-year survival endpoints for characterizing long-term outcomes. Causes of late mortality include progressive tumor growth that is refractory to multiple treatment modalities, secondary malignancies, vascular complications including moyamoya syndrome and stroke, and complications of chronic morbidities including severe obesity.

## 4. Prognostic Factors

### 4.1. Age and Tumor Location

Multiple investigations have identified age below 12 months as an independent negative prognostic factor in pediatric optic pathway gliomas. The concentration of DS cases in this age group aligns with this observation. A younger age at diagnosis correlates with multiple adverse features including larger tumor volumes at presentation, higher likelihood of hypothalamic involvement, increased treatment requirements, and worse functional outcomes. Some studies have identified DS presence as an independent negative prognostic factor even after controlling for age, though other investigations have not confirmed DS as an independent prognostic variable [11]. Anatomical classification systems provide prognostic information. Tumors confined to optic nerves demonstrate the most favorable prognosis. Chiasmatic involvement confers intermediate prognosis. Hypothalamic extension, the defining feature of DS-associated tumors, portends the worst prognosis with the highest rates of progression, vision loss, endocrine complications, and mortality [1,55]. The volume of hypothalamic involvement correlates with endocrine morbidity.

### 4.2. Histology and Molecular Features

Histological grade represents an established prognostic factor. Within DS-associated tumors, the predominance of WHO grade I pilocytic astrocytomas accounts for the relatively favorable survival [1,36]. Pilomyxoid astrocytoma demonstrates higher recurrence rates and worse progression-free survival compared to classic pilocytic astrocytoma [37]. The rare occurrence of higher-grade astrocytomas in the hypothalamic region presenting with DS portends a significantly worse prognosis [39]. Molecular characteristics increasingly inform prognosis. BRAF-KIAA1549 fusion status correlates with favorable outcomes in posterior fossa pilocytic astrocytomas. Some studies suggest BRAF fusion-positive tumors demonstrate a better treatment response and progression-free survival. BRAF V600E mutation status has been associated with worse outcomes in some series. Comprehensive molecular profiling enables refined classification into distinct molecular subgroups with differing clinical behaviors.

### 4.3. NF1 Status and Treatment Response

The prognostic significance of NF1 status in optic pathway gliomas remains controversial, though data specific to DS patients with NF1 are particularly limited given the rarity of DS manifestation in this population. While some studies suggest improved early outcomes in NF1-associated tumors generally, the small numbers of NF1 patients with DS preclude definitive conclusions. Available evidence suggests that NF1-associated optic pathway gliomas more frequently involve bilateral optic nerves and multiple anatomical locations, exhibit slower growth kinetics, and demonstrate lower progression rates during childhood [53,56]. Spontaneous regression or prolonged stability without treatment occurs more commonly in NF1-associated tumors. Given the paucity of data, extrapolation of general NF1-associated optic pathway glioma outcomes to the DS population should be undertaken with caution. Early treatment response represents a strong prognostic indicator for long-term outcomes. Patients achieving objective tumor shrinkage or stabilization with first-line therapy demonstrate superior progression-free and overall survival. Early progression during or immediately following first-line therapy indicates aggressive tumor biology. The number of treatment lines required correlates inversely with survival.

## 5. Quality of Life and Survivorship

The concept of survivorship extends beyond simple survival metrics to encompass multidimensional impacts of disease and treatment. Vision impairment profoundly impacts daily functioning, limiting independence in activities of daily living, mobility, reading ability, and vocational opportunities. Visual rehabilitation services including orientation and mobility training, assistive technology, and adaptive skill development enhance independence.

Endocrine dysfunction necessitates lifelong hormone replacement therapy, regular medical monitoring, and management of associated complications. Cognitive and learning difficulties affect educational attainment, employment prospects, and lifetime earning potential [98]. Psychosocial late effects including depression, anxiety, social isolation, and reduced health-related quality of life occur frequently. The visible effects of disease and treatment may contribute to social difficulties and reduced self-esteem.

Transition from pediatric to adult healthcare systems presents challenges, as coordination of multidisciplinary care becomes more difficult in adult medical systems often lacking expertise in late effects. Structured transition programs facilitate successful transfers of care. Family impacts deserve recognition, as caring for a child with DS affects parental mental health, family functioning, financial stability, and siblings’ well-being.

## 6. Future Directions

Further elucidation of mechanisms underlying DS pathophysiology remains a critical research priority. Advanced metabolic studies incorporating stable isotope tracers, indirect calorimetry, and comprehensive hormonal assessments could clarify whether DS primarily reflects hypermetabolic or anorexic mechanisms. Investigation of hypothalamic neuropeptide systems, neurotransmitter function, and autonomic regulation may identify specific pathways disrupted by tumor involvement.

Identification of biomarkers predicting DS development, treatment response, and long-term outcomes would enable risk-stratified care and personalized treatment selection. Imaging biomarkers derived from advanced MRI techniques could characterize tumor biology and predict progression risk. Volumetric analysis and radiomic approaches show promise for prognostic modeling. Targeted therapies directed against molecular drivers represent a major advance. Expanding access to molecular testing and targeted agents, conducting clinical trials in DS-specific populations, and developing novel targeted approaches hold promise for improving outcomes while reducing toxicity. Ongoing trials investigating MEK inhibitors and BRAF inhibitors specifically in young children with hypothalamic gliomas will inform their role in DS management.

Development of more effective interventions for hypothalamic obesity represents a critical unmet need. Clinical trials evaluating novel pharmacotherapies, behavioral interventions, and potentially bariatric approaches specifically in hypothalamic obesity populations are needed. Strategies to prevent or mitigate treatment-related toxicities including endocrinopathies, cognitive deficits, and vascular complications require investigation. Establishing standardized survivorship care guidelines specific to DS survivors would ensure comprehensive long-term monitoring and management of late effects. Long-term outcome registries and survivorship cohorts enable the study of late effects decades after treatment.

## 7. Conclusions

Diencephalic syndrome represents a rare but clinically significant pediatric disorder demanding heightened clinical awareness for timely diagnosis. The characteristic combination of profound cachexia with preserved linear growth, occurring in infants with normal or minimally reduced caloric intake, should prompt consideration of hypothalamic–chiasmatic pathology. The syndrome’s pathophysiology, while incompletely understood, appears to involve complex dysregulation of hypothalamic function affecting energy homeostasis and growth regulation. Associated low-grade gliomas of the hypothalamic–optic chiasm region represent the predominant underlying etiology, with tumor characteristics and location significantly influencing prognosis.

Despite advances in therapeutic approaches emphasizing chemotherapy to defer radiation in young children, DS-associated tumors confer substantial long-term morbidity. Visual impairment, endocrine dysfunction, and neurological sequelae affect the majority of survivors, necessitating comprehensive multidisciplinary care extending throughout childhood and beyond. The development of hypothalamic obesity in a substantial proportion of survivors represents a particularly challenging late effect significantly impacting quality of life and long-term health.

Survival outcomes vary substantially by age at presentation. While older children demonstrate favorable 5-year overall survival exceeding 90%, infants diagnosed under 1 year experience significantly worse outcomes, with 10-year overall survival of approximately 43%. This dramatic age-dependent difference reflects both more aggressive tumor biology in youngest patients and the particular vulnerability of the developing brain to both disease and treatment effects. The historically high mortality reported in early series has improved with contemporary treatment approaches, though survival in the youngest patients remains a significant challenge. However, high rates of tumor progression necessitate multiple treatment courses in most patients, with associated cumulative toxicity burdens. Extended follow-up reveals an ongoing mortality risk beyond 5 years, emphasizing the chronic nature of this disease and inadequacy of short-term survival endpoints for comprehensive outcome assessment. Prognostic factors including young age, extensive hypothalamic involvement, pilomyxoid histology, and poor treatment response identify high-risk subgroups requiring intensified treatment and surveillance. Molecular characterization increasingly informs risk stratification and enables selection of targeted therapies directed against specific driver alterations.

Enhanced clinical awareness and prompt diagnostic evaluation remain paramount to reducing the risk of irreversible visual loss and improving overall prognosis in this vulnerable population. Multidisciplinary care models integrating oncology, endocrinology, ophthalmology, neurosurgery, nutrition, rehabilitation, and psychosocial services optimize outcomes for children with this complex condition. Structured survivorship programs ensure comprehensive long-term monitoring and management of late effects, supporting survivors’ health, development, and quality of life throughout their lifespan.

## Figures and Tables

**Table 1:** Clinical Features and Management of Diencephalic Syndrome.

Category	Key Features	Clinical Implications
**Core** **Presentation**	• Profound cachexia • Preserved linear growth • Normal/increased appetite • Hyperactivity • Euphoric affect	• Distinguishes from typical FTT • Diagnostic clue when combined • Avg 11-month diagnostic delay
**Visual** **Pathway**	• Nystagmus (majority) • Strabismus • Optic atrophy • Visual field defects	• Often initial symptom • Progressive without treatment • Permanent impairment common
**Neurological**	• Hydrocephalus (40–50%) • Motor abnormalities • Developmental delay • Seizures (15–25%)	• Requires CSF diversion • Variable severity • Impacts long-term function
**Endocrine**	• Central hypothyroidism (60–70%) • Precocious puberty (40–50%) • GH deficiency (20–40%) • DI (20–35%) • Hypothalamic obesity (30–50%)	• Lifelong replacement therapy • Multiple hormone deficiencies • Obesity most challenging • Regular monitoring required
**Associated** **Tumors**	• Pilocytic astrocytoma (70–80%) • Pilomyxoid astrocytoma • Hypothalamic location • BRAF alterations common	• Low-grade histology predominant • Large volume typical • Molecular testing guides therapy • Dissemination rare but reported
**Diagnosis**	• Brain MRI with contrast • Complete neuraxis imaging • Ophthalmologic evaluation • Endocrine assessment	• Essential for all unexplained FTT with normal height • Exclude dissemination • Baseline functional assessment • Guide treatment planning
**Treatment**	• Chemotherapy first-line • Carboplatin–vincristine or vinblastine • MEK/BRAF inhibitors (targeted) • Radiation reserved for salvage (selected cases) • Nutritional support	• Defer radiation in young children • Multiple regimens available • Emerging targeted options • Minimize late effects • Often requires enteral feeding
**Prognosis**	• 5-year OS: 90–100% • 5-year PFS: 10–40% • Significant late morbidity • Visual impairment common • Chronic disease course	• Excellent survival • High progression rates • Lifelong sequelae • Requires long-term follow-up • QOL significantly impacted

FTT: failure to thrive; CSF: cerebrospinal fluid; GH: growth hormone; DI: diabetes insipidus; MRI: magnetic resonance imaging; OS: overall survival; PFS: progression-free survival; QOL: quality of life.

## Data Availability

This review article synthesizes publicly available published literature. All references are cited appropriately to facilitate access to original sources.

## Abbreviations

The following abbreviations are used in this manuscript:

DS	Diencephalic syndrome
GH	Growth hormone
CNS	Central nervous system
PA	Pilocytic Astrocytoma
LGGs	Low grade gliomas

## References

[1] De Martino L., Picariello S., Triarico S., Improda N., Spennato P., Capozza M.A., Grandone A., Santoro C., Cioffi D., Attinà G. (2022). Diencephalic syndrome due to optic pathway gliomas in pediatric patients: An Italian multicenter study. Diagnostics.

[2] Addy D.P., Hudson F.P. (1972). Diencephalic syndrome of infantile emaciation: Analysis of literature and report of further 3 cases. Arch. Dis. Child..

[3] Russell A. (1951). A diencephalic syndrome of emaciation in infancy and childhood. Arch Dis Child..

[4] Kim A., Moon J.S., Yang H.R., Chang J.Y., Ko J.S., Seo J.K. (2015). Diencephalic syndrome: A frequently neglected cause of failure to thrive in infants. Korean J. Pediatr..

[5] van Roessel I.M., Schouten-van Meeteren A.Y.N., Meijer L., Hoving E.W., Bakker B., van Santen H.M. (2022). Transition From Diencephalic Syndrome to Hypothalamic Obesity in Children With Suprasellar Low Grade Glioma: A Case Series. Front. Endocrinol..

[6] Trisciuzzi M.T.S., Riccardi R., Piccardi M., Iarossi G., Buzzonetti L., Dickmann A., Colosimo C., Ruggiero A., Di Rocco C., Falsini B. (2004). A fast visual evoked potential method for functional assessment and follow-up of childhood optic gliomas. Clin. Neurophysiol..

[7] Poussaint T.Y., Barnes P.D., Nichols K., Anthony D.C., Cohen L., Tarbell N.J., Goumnerova L. (1997). Diencephalic syndrome: Clinical features and imaging findings. AJNR Am. J. Neuroradiol..

[8] Burr I., Slonim A., Danish R., Gadoth N., Butler I. (1976). Diencephalic syndrome revisited. J. Pediatr..

[9] Dutton J.J. (1994). Gliomas of the anterior visual pathway. Surv. Ophthalmol..

[10] Listernick R., Louis D.N., Packer R.J., Gutmann D.H. (1997). Optic pathway gliomas in children with neurofibromatosis 1: Consensus statement from the nf1 Optic Pathway Glioma Task Force. Ann. Neurol..

[11] Czyzyk E., Jóźwiak S., Roszkowski M., Schwartz R.A. (2003). Optic pathway gliomas in children with and without neurofibromatosis. J. Child Neurol..

[12] Tang Y., Gutmann D.H. (2023). Neurofibromatosis Type 1-Associated Optic Pathway Gliomas: Current Challenges and Future Prospects. Cancer Manag. Res..

[13] Ruggiero A., Ariano A., Triarico S., Capozza M.A., Ferrara P., Attinà G. (2019). Neonatal pharmacology and clinical implications. Drugs Context.

[14] Nicholson H.S., Kretschmar C.S., Krailo M., Bernstein M., Kadota R., Fort D., Reaman G.H. (2007). Phase 2 study of temozolomide in children and adolescents with recurrent central nervous system tumors: A report from the Children’s Oncology Group. Cancer.

[15] Müller H.L. (2013). Childhood craniopharyngioma. Pituitary.

[16] Santoro C., Perrotta S., Picariello S., Scilipoti M., Cirillo M., Quaglietta L., Cinalli G., Cioffi D., Di Iorgi N., Maghnie M. (2020). Pretreatment endocrine disorders due to optic pathway gliomas in pediatric neurofibromatosis type 1: Multicenter study. J. Clin. Endocrinol. Metab..

[17] Drop S.L.S., Guyda H.J., Colle E. (1980). Inappropriate growth hormone release in the diencephalic syndrome of childhood: Case report and 4 year endocrinological follow-up. Clin. Endocrinol..

[18] Kilday J.-P., Bartels U., Huang A., Barron M., Shago M., Mistry M., Zhukova N., Laperriere N., Dirks P., Hawkins C. (2013). Favorable survival and metabolic outcome for children with diencephalic syndrome using a radiation-sparing approach. J. Neuro-Oncol..

[19] Sklar C.A., Constine L.S. (1995). Chronic neuroendocrinological sequelae of radiation therapy. Int. J. Radiat. Oncol. Biol. Phys..

[20] Agha A., Sherlock M., Brennan S., O’Connor S.A., O’Sullivan E., Rogers B., Faul C., Rawluk D., Tormey W., Thompson C.J. (2005). Hypothalamic-pituitary dysfunction after irradiation of nonpituitary brain tumors in adults. J. Clin. Endocrinol. Metab..

[21] Ogilvy-Stuart A.L., E Clayton P., Shalet S.M. (1994). Cranial irradiation and early puberty. J. Clin. Endocrinol. Metab..

[22] E Merchant T., Goloubeva O., Pritchard D.L., Gaber M., Xiong X., Danish R.K., Lustig R.H. (2002). Radiation dose-volume effects on growth hormone secretion. Int. J. Radiat. Oncol. Biol. Phys..

[23] Gnekow A.K., Falkenstein F., von Hornstein S., Zwiener I., Berkefeld S., Bison B., Warmuth-Metz M., Driever P.H., Soerensen N., Kortmann R.-D. (2012). Long-term follow-up of the multicenter, multidisciplinary treatment study HIT-LGG-1996 for low-grade glioma in children and adolescents of the German Speaking Society of Pediatric Oncology and Hematology. Neuro-Oncology.

[24] Brauner R., Trivin C., Zerah M., Souberbielle J.-C., Doz F., Kalifa C., Sainte-Rose C. (2006). Diencephalic syndrome due to hypothalamic tumor: A model of the relationship between weight and puberty onset. J. Clin. Endocrinol. Metab..

[25] WHO Multicentre Growth Reference Study Group (2006). WHO Child Growth Standards based on length/height, weight and age. Acta Paediatr. Suppl..

[26] Conway M., Ejaz R., Kouzmitcheva E., Savlov D., Rutka J.T., Moharir M. (2016). Child Neurology: Diencephalic syndrome–like presentation of a cervicomedullary brainstem tumor. Neurology.

[27] Webb E.A., Dattani M.T. (2010). Septo-optic dysplasia. Eur. J. Hum. Genet..

[28] Dean H.J., Kellett J.G., Bala R.M., Guyda H.J., Bhaumick B., Posner B.I., Friesen H.G. (1982). The effect of growth hormone treatment on somatomedin levels in a patient with dience-phalic syndrome. J. Clin. Endocrinol. Metab..

[29] Albert B.D., Spolidoro G.C., Mehta N.M. (2021). Metabolism and energy prescription in critically ill children. Minerva Anestesiol..

[30] Sherwood L.M., Parris E.E., Cahill G.F. (1970). Starvation in man. N. Engl. J. Med..

[31] Timper K., Brüning J.C. (2017). Hypothalamic circuits regulating appetite and energy homeostasis: Pathways to obesity. Dis. Model. Mech..

[32] Cummings D.E., Weigle D.S., Frayo R.S., Breen P.A., Ma M.K., Dellinger E.P., Purnell J.Q. (2002). Plasma ghrelin levels after diet-induced weight loss or gastric bypass surgery. N. Engl. J. Med..

[33] Landsberg L., Young J.B. (1978). Fasting, feeding and regulation of the sympathetic nervous system. N. Engl. J. Med..

[34] Tisdale M.J. (2002). Cachexia in cancer patients. Nat. Rev. Cancer.

[35] Perilongo G., Carollo C., Salviati L., Murgia A., Pillon M., Basso G., Gardiman M., Laverda A. (1997). Diencephalic syndrome and disseminated juvenile pilocytic astrocytomas of the hypothalamic-optic chiasm region. Cancer.

[36] Tihan T., Fisher P.G., Kepner J.L., Godfraind C., McComb R.D., Goldthwaite P.T., Burger P.C. (1999). Pediatric astrocytomas with monomorphous pilomyxoid features and a less favorable outcome. J. Neuropathol. Exp. Neurol..

[37] Qaddoumi I., Orisme W., Wen J., Santiago T., Gupta K., Dalton J.D., Tang B., Haupfear K., Punchihewa C., Easton J. (2016). Genetic alterations in uncommon low-grade neuroepithelial tumors: BRAF, FGFR1, and MYB mutations occur at high frequency and align with morphology. Acta Neuropathol..

[38] Sturm D., Pfister S.M., Jones D.T. (2017). Pediatric gliomas: Current concepts on diagnosis, biology, and clinical management. J. Clin. Oncol..

[39] Perrone M.G., Ruggiero A., Centonze A., Carrieri A., Ferorelli S., Scilimati A. (2021). Diffuse Intrinsic Pontine Glioma (DIPG): Breakthrough and Clinical Perspective. Curr. Med. Chem..

[40] Louis D.N., Perry A., Reifenberger G., Von Deimling A., Figarella-Branger D., Cavenee W.K., Ohgaki H., Wiestler O.D., Kleihues P., Ellison D.W. (2016). The 2016 World Health Organization Classification of Tumors of the Central Nervous System: A summary. Acta Neuropathol..

[41] Jones D.T.W., Kieran M.W., Bouffet E., Alexandrescu S., Bandopadhayay P., Bornhorst M., Ellison D., Fangusaro J., Fisher M.J., Foreman N. (2018). Pediatric low-grade gliomas: Next biologically driven steps. Neuro-Oncol..

[42] Jones D.T., Kocialkowski S., Liu L., Pearson D.M., Bäcklund L.M., Ichimura K., Collins V.P. (2008). Tandem duplication producing a novel oncogenic BRAF fusion gene defines the majority of pilocytic astrocytomas. Cancer Res..

[43] Schindler G., Capper D., Meyer J., Janzarik W., Omran H., Herold-Mende C., Schmieder K., Wesseling P., Mawrin C., Hasselblatt M. (2011). Analysis of BRAF V600E mutation in 1,320 nervous system tumors reveals high mutation frequencies in pleomorphic xanthoastrocytoma, ganglioglioma and extra-cerebellar pilocytic astrocytoma. Acta Neuropathol..

[44] Jones D.T.W., Hutter B., Jäger N., Korshunov A., Kool M., Warnatz H.-J., Zichner T., Lambert S.R., Ryzhova M., Quang D.A. (2013). Recurrent somatic alterations of FGFR1 and NTRK2 in pilocytic astrocytoma. Nat. Genet..

[45] Fangusaro J., Onar-Thomas A., Poussaint T.Y., Wu S., Ligon A.H., Lindeman N., Banerjee A., Packer R.J., Kilburn L.B., Goldman S. (2019). Selumetinib in paediatric patients with BRAF-aberrant or neurofibromatosis type 1-associated recurrent, refractory, or progressive low-grade glioma: A multicentre, phase 2 trial. Lancet Oncol..

[46] Bouffet E., Hansford J.R., Garrè M.L., Hara J., Plant-Fox A., Aerts I., Hargrave D.R. (2023). Dabrafenib plus Trametinib in Pediatric Glioma with BRAF V600 Mutations. N. Engl. J. Med..

[47] Talloa D., Triarico S., Agresti P., Mastrangelo S., Attinà G., Romano A., Maurizi P., Ruggiero A. (2022). BRAF and MEK Targeted Therapies in Pediatric Central Nervous System Tumors. Cancers.

[48] Zhang M., Chen T., Zhong Y. (2021). Demographic and prognostic factors of optic nerve astrocytoma: A retrospective study of surveillance, epidemiology, and end results (SEER). BMC Cancer.

[49] D’AMico A., Rosano C., Pannone L., Pinna V., Assunto A., Motta M., Ugga L., Daniele P., Mandile R., Mariniello L. (2021). Clinical variability of neurofibromatosis 1: A modifying role of cooccurring PTPN11 variants and atypical brain MRI findings. Clin. Genet..

[50] Listernick R., Ferner R.E., Liu G.T., Gutmann D.H. (2007). Optic pathway gliomas in neurofibromatosis-1: Controversies and recommendations. Ann. Neurol..

[51] Listernick R., Charrow J., Greenwald M., Mets M. (1994). Natural history of optic pathway tumors in children with neurofibromatosis type 1: A longitudinal study. J. Pediatr..

[52] Blazo M., Lewis R., Chintagumpala M., Frazier M., McCluggage C., Plon S. (2004). Outcomes of systematic screening for optic pathway tumors in children with neurofibromatosis type 1. Am. J. Med Genet. Part A.

[53] Cavicchiolo M.E., Opocher E., Daverio M., Bendini M., Viscardi E., Bisogno G., Perilongo G., Da Dalt L. (2013). Diencephalic syndrome as sign of tumor progression in a child with neurofibromatosis type 1 and optic pathway glioma: A case report. Child’s Nerv. Syst..

[54] Taylor T., Jaspan T., Milano G., Gregson R., Parker T., Ritzmann T., Benson C., Walker D. (2008). Radiological classification of optic pathway gliomas: Experience of a modified functional classification system. Br. J. Radiol..

[55] Lu Y., Xu M., Chen X., Xu H., Sun N., Weisgerber K.E., Bai R.-Y. (2025). Neurofibromatosis Type 1: Genetic Mechanisms and Advances in Therapeutic Innovation. Cancers.

[56] Packer R.J., Ater J., Allen J., Phillips P., Geyer R., Nicholson H.S., Jakacki R., Kurczynski E., Needle M., Finlay J. (1997). Carboplatin and vincristine chemotherapy for children with newly diagnosed progressive low-grade gliomas. J. Neurosurg..

[57] Swiatek V.M., Stein K.-P., Cukaz H.B., Rashidi A., Skalej M., Mawrin C., Sandalcioglu I.E., Neyazi B. (2021). Spinal intramedullary schwannomas—report of a case and extensive review of the literature. Neurosurg. Rev..

[58] Komotar R.J., Mocco J., Jones J.E., Zacharia B.E., Tihan T., Feldstein N.A., Anderson R.C.E. (2005). Pilomyxoid astrocytoma: Diagnosis, prognosis, and management. Neurosurg. Focus.

[59] Farche M.K., O Fachinetti N., da Silva L.R., Matos L.A., Appenzeller S., Cendes F., Reis F. (2022). Revisiting the use of proton magnetic resonance spectroscopy in distinguishing between primary and secondary malignant tumors of the central nervous system. Neuroradiol. J..

[60] Kahalley L.S., Ris M.D., Grosshans D.R., Okcu M.F., Paulino A.C., Chintagumpala M., Moore B.D., Guffey D., Minard C.G., Stancel H.H. (2016). Comparing intelligence quotient change after treatment with proton versus photon radiation therapy for pediatric brain tumors. J. Clin. Oncol..

[61] Dodge H.W., Love J.G., Craig W.M., Dockerty M.B., Kearns T.P., Holman C.B., Hayles A.B. (1958). Gliomas of the optic nerves. AMA Arch. Neurol. Psychiatry.

[62] Friedman J.M., Adam M.P., Bick S., Mirzaa G.M., Pagon R.A., Wallace S.E., Amemiya A. (2025). Neurofibromatosis 1.

[63] von Hornstein S., Kortmann R., Pietsch T., Emser A., Warmuth-Metz M., Soerensen N., Straeter R., Graf N., Thieme B., Gnekow A.K. (2011). Impact of chemotherapy on disseminated low-grade glioma in children and adolescents: Report from the HIT-LGG 1996 trial. Pediatr. Blood Cancer.

[64] Lassaletta A., Scheinemann K., Zelcer S.M., Hukin J., Wilson B.A., Jabado N., Carret A.S., Lafay-Cousin L., Larouche V., Hawkins C.E. (2016). Phase II weekly vinblastine for chemotherapy-naïve children with progressive low-grade glioma: A Canadian Pediatric Brain Tumor Consortium study. J. Clin. Oncol..

[65] Campagna M., Opocher E., Viscardi E., Calderone M., Severino S.M., Cermakova I., Perilongo G. (2010). Optic pathway glioma: Long-term visual outcome in children without neurofibromatosis type-1. Pediatr. Blood Cancer.

[66] Fisher M.J., Loguidice M., Gutmann D.H., Listernick R., Ferner R.E., Ullrich N.J., Packer R.J., Tabori U., Hoffman R.O., Ardern-Holmes S.L. (2012). Visual outcomes in children with neurofibromatosis type 1-associated optic pathway glioma following chemotherapy: A multicenter retrospective analysis. Neuro-Oncol..

[67] Lazzareschi I., Ruggiero A., Riccardi R., Attinà G., Colosimo C., Lasorella A. (2002). Hypersensitivity reactions to carboplatin in children. J. Neuro-Oncol..

[68] Packer R.J., Pfister S., Bouffet E., Avery R., Bandopadhayay P., Bornhorst M., Bowers D.C., Ellison D., Fangusaro J., Foreman N. (2017). Pediatric low-grade gliomas: Implications of the biologic era. Neuro-Oncol..

[69] Bouffet E., Jakacki R., Goldman S., Hargrave D., Hawkins C., Shroff M., Hukin J., Bartels U., Foreman N., Kellie S. (2012). Phase II study of weekly vinblastine in recurrent or refractory pediatric low-grade glioma. J. Clin. Oncol..

[70] Lafay-Cousin L., Holm S., Qaddoumi I., Nicolin G., Bartels U., Tabori U., Huang A., Bouffet E. (2005). Weekly vinblastine in pediatric low-grade glioma patients with carboplatin allergic reaction. Cancer.

[71] Mirow C., Pietsch T., Berkefeld S., Kwiecien R., Warmuth-Metz M., Falkenstein F., Diehl B., von Hornstein S., Gnekow A.K. (2014). Children <1 year show an inferior outcome when treated according to the traditional LGG treatment strategy: A report from the german multicenter trial HIT-LGG 1996 for children with low grade glioma (LGG). Pediatr. Blood Cancer.

[72] Geyer J.R., Sposto R., Jennings M., Boyett J.M., Axtell R.A., Breiger D., Broxson E., Donahue B., Finlay J.L., Goldwein J.W. (2005). Multiagent chemotherapy and deferred radiotherapy in infants with malignant brain tumors: A report from the Children’s Cancer Group. J. Clin. Oncol..

[73] Sofia R., Melita V., De Vita A., Ruggiero A., Romano A., Attinà G., Birritella L., Lamendola P., Lombardo A., Lanza G.A. (2021). Cardiac Surveillance for Early Detection of Late Subclinical Cardiac Dysfunction in Childhood Cancer Survivors After Anthracycline Therapy. Front. Oncol..

[74] Jenkin D., Angyalfi S., Becker L., Berry M., Buncic R., Chan H., Doherty M., Drake J., Greenberg M., Hendrick B. (1993). Optic glioma in children: Surveillance, resection, or irradiation?. Int. J. Radiat. Oncol. Biol. Phys..

[75] Armstrong G.T., Liu Q., Yasui Y., Huang S., Ness K.K., Leisenring W., Hudson M.M., Donaldson S.S., King A.A., Stovall M. (2009). Long-term outcomes among adult survivors of childhood central nervous system malignancies in the Childhood Cancer Survivor Study. JNCI J. Natl. Cancer Inst..

[76] Sethi R.V., Shih H.A., Yeap B.Y., Mouw K.W., Petersen R., Kim D.Y., Munzenrider J.E., Grabowski E., Rodriguez-Galindo C., Yock T.I. (2014). Second nonocular tumors among survivors of retinoblastoma treated with contemporary photon and proton radiotherapy. Cancer.

[77] Mehta V., Chapman A., McNeely P.D., Walling S., Howes W.J. (2002). Latency between symptom onset and diagnosis of pediatric brain tumors: An Eastern Canadian geographic study. Neurosurgery.

[78] Kelly J.P., Weiss A.H. (2013). Detection of tumor progression in optic pathway glioma with and without neurofibromatosis type 1. Neuro-Oncol..

[79] Via P.D., Opocher E., Pinello M.L., Calderone M., Viscardi E., Clementi M., Battistella P.A., Laverda A.M., Da Dalt L., Perilongo G. (2007). Visual outcome of a cohort of children with neurofibromatosis type 1 and optic pathway glioma followed by a pediatric neuro-oncology program. Neuro-Oncol..

[80] Dattani M., Preece M. (2004). Growth hormone deficiency and related disorders: Insights into causation, diagnosis, and treatment. Lancet.

[81] Ergun-Longmire B., Mertens A.C., Mitby P., Qin J., Heller G., Shi W., Yasui Y., Robison L.L., Sklar C.A. (2006). Growth hormone treatment and risk of second neoplasms in the childhood cancer survivor. J. Clin. Endocrinol. Metab..

[82] Mackenzie S., Craven T., Gattamaneni H.R., Swindell R., Shalet S.M., Brabant G. (2011). Long-term safety of growth hormone replacement after CNS irradiation. J. Clin. Endocrinol. Metab..

[83] Carel J.-C., Léger J. (2008). Clinical practice. Precocious puberty. N. Engl. J. Med..

[84] Lazar L., Padoa A., Phillip M. (2007). Growth pattern and final height after cessation of gonadotropin-suppressive therapy in girls with central sexual precocity. J. Clin. Endocrinol. Metab..

[85] Bondy C.A. (2007). Turner Syndrome Study Group. Care of girls and women with Turner syndrome: A guideline of the Turner Syn-drome Study Group. J. Clin. Endocrinol. Metab..

[86] Hahner S., Ross R.J., Arlt W., Bancos I., Burger-Stritt S., Torpy D.J., Quinkler M. (2021). Adrenal insufficiency. Nat. Rev. Dis. Primers.

[87] Di Iorgi N., Napoli F., Allegri A.E.M., Olivieri I., Bertelli E., Gallizia A., Rossi A., Maghnie M. (2012). Diabetes insipidus—Diagnosis and management. Horm. Res. Paediatr..

[88] Robertson G.L. (2016). Diabetes insipidus: Differential diagnosis and management. Best Pr. Res. Clin. Endocrinol. Metab..

[89] Roth C.L., Elfers C., Gebhardt U., Müller H.L., Reinehr T. (2013). Brain-derived neurotrophic factor and its relation to leptin in obese children before and after weight loss. Metabolism.

[90] Holmer H., Pozarek G., Wirfält E., Popovic V., Ekman B., Björk J., Erfurth E.-M. (2010). Reduced energy expenditure and impaired feeding-related signals but not high energy intake reinforces hypothalamic obesity in adults with childhood onset craniopharyngioma. J. Clin. Endocrinol. Metab..

[91] Lustig R.H., Post S.R., Srivannaboon K., Rose S.R., Danish R.K., Burghen G.A., Merchant T.E. (2003). Risk factors for the development of obesity in children surviving brain tumors. J. Clin. Endocrinol. Metab..

[92] Cefalo M.G., Maurizi P., Arlotta A., Scalzone M., Attinà G., Ruggiero A., Riccardi R. (2010). Hepatic veno-occlusive disease: A chemotherapy-related toxicity in children with ma-lignancies. Paediatr. Drugs.

[93] Ruggiero A., Barone G., Liotti L., Chiaretti A., Lazzareschi I., Riccardi R. (2007). Safety and efficacy of fentanyl administered by patient controlled analgesia in children with cancer pain. Support. Care Cancer.

[94] Posteraro B., Bruno S., Boccia S., Ruggiero A., Sanguinetti M., Spica V.R., Ricciardi G., Fadda G. (2004). *Candida parapsilosis* bloodstream infection in pediatric oncology patients: Results of an epidemiologic investigation. Infect. Control. Hosp. Epidemiol..

[95] Hamilton J.K., Conwell L.S., Syme C., Ahmet A., Jeffery A., Daneman D. (2011). Hypothalamic obesity following craniopharyngioma surgery: Results of a pilot trial of combined diazoxide and metformin therapy. Int. J. Pediatr. Endocrinol..

[96] Romano A., Triarico S., Rinninella E., Natale L., Brizi M.G., Cintoni M., Raoul P., Maurizi P., Attinà G., Mastrangelo S. (2022). Clinical Impact of Nutritional Status and Sarcopenia in Pediatric Patients with Bone and Soft Tissue Sarcomas: A Pilot Retrospective Study (SarcoPed). Nutrients.

[97] Snook A.G., A Arnadottir S., Forbes R. (2023). A survey of patient education practices and perceptions of physiotherapists: A mixed methods study. Physiother. Theory Pract..

[98] Mulhern R.K., Merchant T.E., Gajjar A., Reddick W.E., Kun L.E. (2004). Late neurocognitive sequelae in survivors of brain tumours in childhood. Lancet Oncol..

[99] Butler R.W., Haser J.K. (2006). Neurocognitive effects of treatment for childhood cancer. Ment. Retard. Dev. Disabil. Res. Rev..

[100] Edelstein K., Spiegler B.J., Fung S., Panzarella T., Mabbott D.J., Jewitt N., D’Agostino N.M., Mason W.P., Bouffet E., Tabori U. (2011). Early aging in adult survivors of childhood medulloblastoma: Long-term neurocognitive, functional, and physical outcomes. Neuro-Oncol..

[101] Conklin H.M., Khan R.B., Reddick W.E., Helton S., Brown R., Howard S.C., Bonner M., Christensen R., Wu S., Xiong X. (2007). Acute neurocognitive response to methylphenidate among survivors of childhood cancer: A randomized, double-blind, cross-over trial. J. Pediatr. Psychol..

[102] Schulte F., Barrera M. (2010). Social competence in childhood brain tumor survivors: A comprehensive review. Support. Care Cancer.

[103] Ellenberg L., Liu Q., Gioia G., Yasui Y., Packer R.J., Mertens A., Donaldson S.S., Stovall M., Kadan-Lottick N., Armstrong G. (2009). Neurocognitive status in long-term survivors of childhood CNS malignancies: A report from the Childhood Cancer Survivor Study. Neuropsychology.

[104] Mabbott D.J., Spiegler B.J., Greenberg M.L., Rutka J.T., Hyder D.J., Bouffet E. (2005). Serial evaluation of academic and behavioral outcome after treatment with cranial radiation in childhood. J. Clin. Oncol..

